# Proteomic profiling of sweat in patients with cystic fibrosis provides new insights into epidermal homoeostasis

**DOI:** 10.1002/ski2.161

**Published:** 2022-11-25

**Authors:** Matthieu Cornet, Thao Nguyen‐Khoa, Mairead Kelly‐Aubert, Vincent Jung, Frédérique Chedevergne, Muriel Le Bourgeois, Laura Aoust, Kévin Roger, Chiara Ida Guerrera, Isabelle Sermet‐Gaudelus

**Affiliations:** ^1^ Institut Necker Enfants Malades INSERM U1151 Paris France; ^2^ Center for Computational Biology Mines ParisTech PSL Research University Paris France; ^3^ Institut Curie Paris France; ^4^ INSERM U900 Paris France; ^5^ Laboratory of Biochemistry Hôpital Universitaire Necker Enfants Malades AP‐HP Centre Paris France; ^6^ Université Paris Cité Paris France; ^7^ Proteomics Platform Necker Université Paris Cité Structure Fédérative de Recherche Necker (SFR Necker, INSERM US24/CNRS UAR3633) Paris France; ^8^ Centre de Référence Maladies Rares Mucoviscidose et Maladies Apparentées Hôpital Necker Enfants Malades AP‐HP Centre Paris Cité Paris France; ^9^ European Respiratory Network ERN Lung Frankfurt Germany

## Abstract

**Background:**

A high proportion of patients with Cystic Fibrosis (CF) also present the rare skin disease aquagenic palmoplantar keratoderma. A possible link between this condition and absence of a functional CF Transmembrane conductance Regulator protein in the sweat acinus and collecting duct remains unknown.

**Methods:**

In‐depth characterization of sweat proteome profiles was performed in 25 CF patients compared to 12 healthy controls. A 20 μL sweat sample was collected after pilocarpine iontophoresis and liquid chromatography tandem mass spectrometry (LC‐MS/MS) proteomic analysis was performed.

**Results:**

Sweat proteome profile of CF patients was significantly different from that of healthy subjects with 57 differentially expressed proteins. Cystic Fibrosis sweat proteome was characterized by an increase in 25 proteins including proteases (Kallikrein 7 and 13, Phospholipase B domain containing 1, Cathepsin A L2 and B, Lysosomal Pro‐X carboxypeptidase); proinflammatory proteins (Annexin A2, Chitinase‐3‐like protein 1); cytochrome c and transglutaminases. Thirty‐two proteins were downregulated in CF sweat including proteases (Elastase 2), antioxidative protein FAM129 B; membrane‐bound transporter SLC6A14 and regulator protein Sodium‐hydrogen antiporter 3 regulator 1.

**Conclusion:**

This study is the first to report in‐depth characterization of endogenous peptides in CF sweat and could help understand the complex physiology of the sweat gland. The proteome profile highlights the unbalanced proteolytic and proinflammatory activity of sweat in CF. These results also suggest a defect in pathways involved in skin barrier integrity in CF patients. Sweat proteome profile could prove to be a useful tool in the context of personalized medicine in CF.

## Graphical abstract

We performed in‐depth profiling of sweat protein composition on 25 patients with Cystic Fibrosis (CF), and 12 non‐ individuals. Our results provide evidence for an unbalanced proteolytic activity and inflammation of CF sweat and alteration of other signaling pathways which may alter integrity of the epidermal skin barrier. This suggests that CFTR is involved in the fine tuning of the proteolytic and inflammatory balance in skin, and indirectly in the integrity of the epidermis barrier.

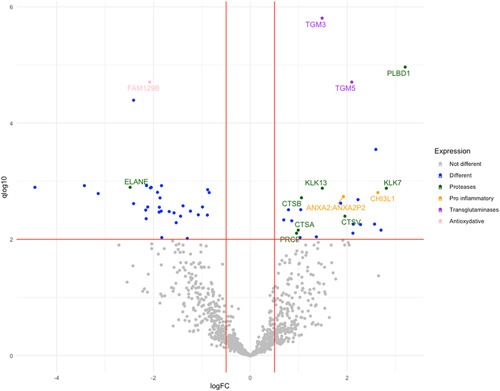

1

**What is already known about this topic?**

- Although sweat is a reliable, non‐invasive, and easy to collect biofluid, sweat proteome profiles as a source of biomarkers has been studied in relatively few diseases.

**What does this study add?**

- Our study is the first to attempt a comparative analysis of sweat proteome profiles between Cystic Fibrosis (CF) patients and controls and reveals a unique subset of differentially expressed proteins. The results provide new insights to epidermal homoeostasis in CF.

**What is the translational message?**

- Sweat proteome profiles could serve as convenient tools in CF for diagnosis or personalized therapeutic interventions.

## 2 INTRODUCTION

One of the hallmarks of Cystic Fibrosis (CF) is high Chloride (Cl^−^) sweat concentration due to the absence of the CF Transmembrane conductance Regulator (CFTR) protein in the sweat acinus and collecting duct.^1^ Patients with CF (pwCF) can display aquagenic palmoplantar keratoderma (AKP), characterized by exaggerated and early wrinkling of the skin after brief immersion in water associated with tingling and pain sensation.^2^ This condition is probably underestimated as AKP is observed in 41%–84% of CF patients.^3^, ^4^ The physiopathology of AKP and its relationship to CFTR dysregulation is not yet fully elucidated. This condition has been recently reported to be improved by CFTR modulators in CF patients which relates this skin phenotype to CFTR defects.^5^ To gain further insight into the pathophysiology of this rare skin disorder, we performed in‐depth analysis of the sweat proteome of pwCF compared to non‐CF individuals.

## 3 PATIENTS AND METHODS

### 3.1 Patients

We enroled 25 pwCF, and 12 non‐CF individuals (WT). Patients' genotype was classified according to genetic mechanism of the variant (class 1: nonsense, deletion or severe splicing; class 2: misfolding; class 3: activation defect; class 4: conductance defect; class 5: splicing with residual function).^1^ All participants signed an informed consent form for sweat collection and analysis (no. Eudract 2016‐A00309‐42; Comité de Protection des Personnes IDF2).

Clinical status of the patient at the study visit was assessed by pancreatic status, microbiology of sputum and Respiratory Volume in 1 s (FEV1), expressed as percentage predicted (ppFEV1). Sweat induction was performed by pilocarpine iontophoresis, as previously reported.^6^ Sweat was collected using the Wescor Macroduct (ELITech, Puteaux, France) and chloride concentration was assessed by coulometry (chloridometer 926S Sherwood, Servilab, Le Mans, France). Twenty μl of remaining sweat sample was used for proteomic analysis.

### 3.2 Sample preparation for liquid chromatography mass spectrometry (LC‐MS/MS) analysis

Twenty μL of sweat were processed in the S‐TrapTM micro spin column (Protifi, Hutington, USA) for digestion according to manufacturer's instructions. Briefly, sodium dodecyl sulphate (SDS) was added to a final concentration of 5% and samples were reduced with 20 mM tris(2‐carboxyethyl)phosphine (TCEP) and alkylated with 50 mM chloracetamide (CAA) for 15 min at room temperature. Aqueous phosphoric acid was then added to a final concentration of 1.2% followed by the addition of six volumes of S‐Trap binding buffer (90% aqueous methanol, 100 mM triethylammonium bicarbonate buffer (TEAB), pH 7.1). Mixtures were then loaded on S‐Trap columns. Six washing steps were performed for thorough SDS elimination. Samples were digested with 0.8 μg of trypsin (Promega) at 47°C for 1h30. After elution, peptides were vacuum dried and resuspended in 30 μL of 2% acetonitrile (ACN), 0.1% formic acid (FA) in HPLC‐grade water prior to mass spectrometry (MS) analysis. The concentration of the peptide mixtures was determined using a Nanodrop 2000 from Labtech France.

### 3.3 LC‐MS/MS analysis

For each sample, 400 ng of peptides were injected on a nanoelute (Bruker Daltonics, Germany) high‐performance liquid chromatography system coupled to a timsTOF Pro (Bruker Daltonics, Germany) mass spectrometer. High‐performance liquid chromatography separation (Solvent A: 0.1% FA in water, 2% ACN; Solvent B: 0.1% FA in ACN) was carried out at 250 nL/min using a packed emitter column (C18, 25 cm × 75 μm 1.6 μm) (Ion Optics, Australia) with a gradient elution (2%–11% solvent B during 19 min; 11%–16% for 7 min; 16%–25% for 4 min; 25%–80% for 3 min and finally 80% for 7 min to wash the column). Mass‐spectrometric data were acquired using the parallel accumulation serial fragmentation (PASEF) acquisition method. The measurements were carried out over the *m*/*z* range from 100 to 1700 Th. The ion mobilities values ranged from 0.8 to 1.3 V s/cm2 (1/k0). The total cycle time was set to 1.16 s and the number of PASEF MS/MS scans was set to 10. For low sample amounts, the total cycle time was set to 1.88 s. QCs, consisting in 10 ng of Hela total cell lysates were run before, after and during the acquisition of the series.

### 3.4 Data analysis

The data were analyzed using MaxQuant version 2.0.17.0 and searched with Andromeda search engine against the UniProtKB/Swiss‐Prot Homo sapiens database (release 02‐2021, 20 396 entries). To search parent mass and fragment ions, we set a mass deviation of 3 and 20 ppm respectively. The minimum peptide length was set to 7 amino acids and strict specificity for trypsin cleavage was required, allowing up to two missed cleavage sites. Carbamidomethylation (Cys) was set as fixed modification, whereas oxidation (Met) and N‐term acetylation were set as variable modifications. The false discovery rates at the protein and peptide level were set to 1%. Scores were calculated in MaxQuant as described previously.^7^ The reverse and common contaminants hits were removed from MaxQuant output. Proteins were quantified according to the MaxQuant label‐free algorithm using lable free quantification intensities, providing a label‐free normalization; protein quantification was obtained using at least 2 peptides per protein. Match between runs was allowed.

Data filtering and imputation was performed using the Prostar Software. Proteins were retained in the analysis if they were detected in at least 70% of the patients in at least one group. Statistical analyses were conducted in R. Data in pwCF and WT conditions were compared by Student *t*‐test. Benjamini‐Hochberg corrections were applied to account for multiple testing. A *q*‐value <0.01 combined with a log2 fold change >0.5 was considered statistically significant.

## 4 RESULTS

### 4.1 Population

We enroled 25 pwCF, mean age 9.1(6.3) years, all carrying 2 CF causing variants of the *CFTR* gene (11 homozygous p.Phe508del (F508del thereafter); 7 F508del compound heterozygotes; 7 carrying nonsense, splicing mutations or large deletion variants) (Table 1). Patient 13 carried the rare missense variant p.Phe191Val (F191 V) in *trans* of F508del and had a normal chloride sweat concentration at 22 mmol/L. However, *β*‐adrenergic sweat secretion rate was in the CF range, providing insight for a genotype with minimal CFTR activity.^8^ Patient 20 carried the 2789 + 5G > A splice site mutation, known to be associated with a small amount of normally spliced transcripts.^9^ All patients were at steady state at the study visit. Three patients (patient 13, 20, 25) were pancreatic sufficient. Patients 2, 3, and 21 had chronic sputum colonization with *Pseudomonas*.*aeruginosa* or *Alcaligenes*.*spp* and were treated with inhaled antibiotics. The 12 non‐CF individuals (WT) had a mean age of 12.5 (10.8) years and a normal sweat test.

**TABLE 1:** Patients characteristics at the moment of sweat collection

	Age (years)	Sex	Genotype		Genotype classification	Sweat test (mmol/L)	Pancreatic status	FEV1 pp	Bronchial colonization
Patients									
1	13	M	F508del (a)	F508del (a)	2/2	81	PI	104	*S*.*aureus*
2	16	M	444delA	444delA	1/1	107	PI	ND	*S*.*aureus*, *Alcaligenes*
3	13	M	F508del(a)	L558S	2/2	105	PI	33	*P*.*aeruginosa*, *Alcaligenes* spp
4	12	F	F508del(a)	F508del(a)	2/2	110	PI	116	*S*.*aureus*
5	13	F	G542X(c)	G542X(c)	1/1	118	PI	90	*S*.*aureus*, *Alcaligenes* spp
6	3	M	G542X(c)	G542X(c)	1/1	114	PI	ND	*P*.*aeruginosa*
7	17	M	F508del(a)	F508del(a)	2/2	120	PI	129	*S*.*aureus*
8	19	F	F508del(a)	F508del(a)	2/2	69	PI	96	*S*.*aureus*
9	0.1	M	F508del(a)	W1282X(c)	2/1	95	PI	ND	None detected
10	3	M	Y275X(c)	S466X(c)	1/1	126	PI	ND	*S*.*aureus*
11	0.1	F	F508del(a)	del exons 2‐3(b)	2/1	102	PI	ND	None detected
12	13	F	F508del(a)	H199Y(a)	2/2	101	PI	109	*S*.*aureus*
13	4	M	F508del(a)	F191 V(a)	2/2	22	PS	100	None detected
14	3	F	1717‐1G>A(d)	1717‐1G>A(d)	1/1	109	PI	ND	None detected
15	5	F	F508del(a)	F508del(a)	2/2	110	PI	ND	None detected
16	13	M	F508del(a)	F508del(a)	2/2	105	PI	93	*S*.*aureus*
17	10	M	F508del(a)	F508del(a)	2/2	104	PI	129	*S*.*Maltophilia*
18	1.2	M	I507del(a)	Del Exon 17B (b)	2/1	111	PI	ND	None detected
19	0.1	M	F508del(a)	F508del(a)	2/2	96	PI	ND	None detected
20	15	M	2789 + 5G > A	3120 + 1G > A	5/1	103	PS	59	*S*.*aureus*
21	17	M	F508del(a)	2143delT	2/1	106	PI	41	*P*.*aeruginosa*, *Alcaligenes* spp
22	0.1	M	F508del(a)	F508del(a)	2/2	98	PI	ND	*S*.*aureus*
23	8	F	F508del(a)	F508del(a)	2/2	114	PI	116	None detected
24	14	F	F508del(a)	F508del(a)	2/2	92	PI	108	*S*.*aureus*
25	13	F	F508del(a)	R347H(b)	2/2	49	PS	100	None detected
Non‐CF control subjects									
1	25	M	NA	NA	NA	25	NA	NA	NA
2	0.8	M	NA	NA	NA	11	NA	NA	NA
3	14	M	NA	NA	NA	<10	NA	NA	NA
4	24	F	NA	NA	NA	10	NA	NA	NA
5	0.4	F	NA	NA	NA	<10	NA	NA	NA
6	8	F	NA	NA	NA	17	NA	NA	NA
7	6	F	NA	NA	NA	17	NA	NA	NA
8	27	F	NA	NA	NA	12	NA	NA	NA
9	25	M	NA	NA	NA	19	NA	NA	NA
10	18	M	NA	NA	NA	12	NA	NA	NA
11	0.8	M	NA	NA	NA	11	NA	NA	NA
12	3	M	NA	NA	NA	12	NA	NA	NA

Abbreviations: CF, Cystic Fibrosis; NA, not adapted; ND, not determined; PI, pancreatic insufficient; PS, pancreatic sufficient.

### 4.2 Proteomic analysis

Protein concentration ranged between 0.25 and 0.70 μg/μL of sweat with no difference between pwCF and WT. Nine hundred ninety‐one proteins were retained for the analysis. Data are available via ProteomeXchange with identifier PXD032894.

**FIGURE 1:**
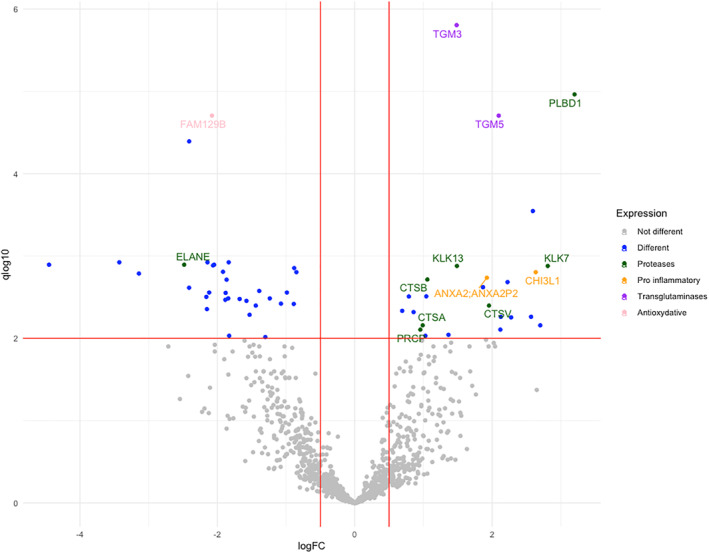
Volcano plot of differentially expressed proteins between patients with cystic Fibrosis (CF) (patients with CF (pwCF)) and non‐CF individuals (WT). Thresholds of 0.5 for log2 Fold Change (FC) in abscissa, and of 0.01 for *q* values (expressed as‐log10) in ordinate, are used for significancy and plotted in green. Differentially expressed proteins are plotted in colour (Proteases: green; proinflammatory proteins: orange; transglutaminases: purple; antioxidative proteins: pink; others: blue)

**FIGURE 2:**
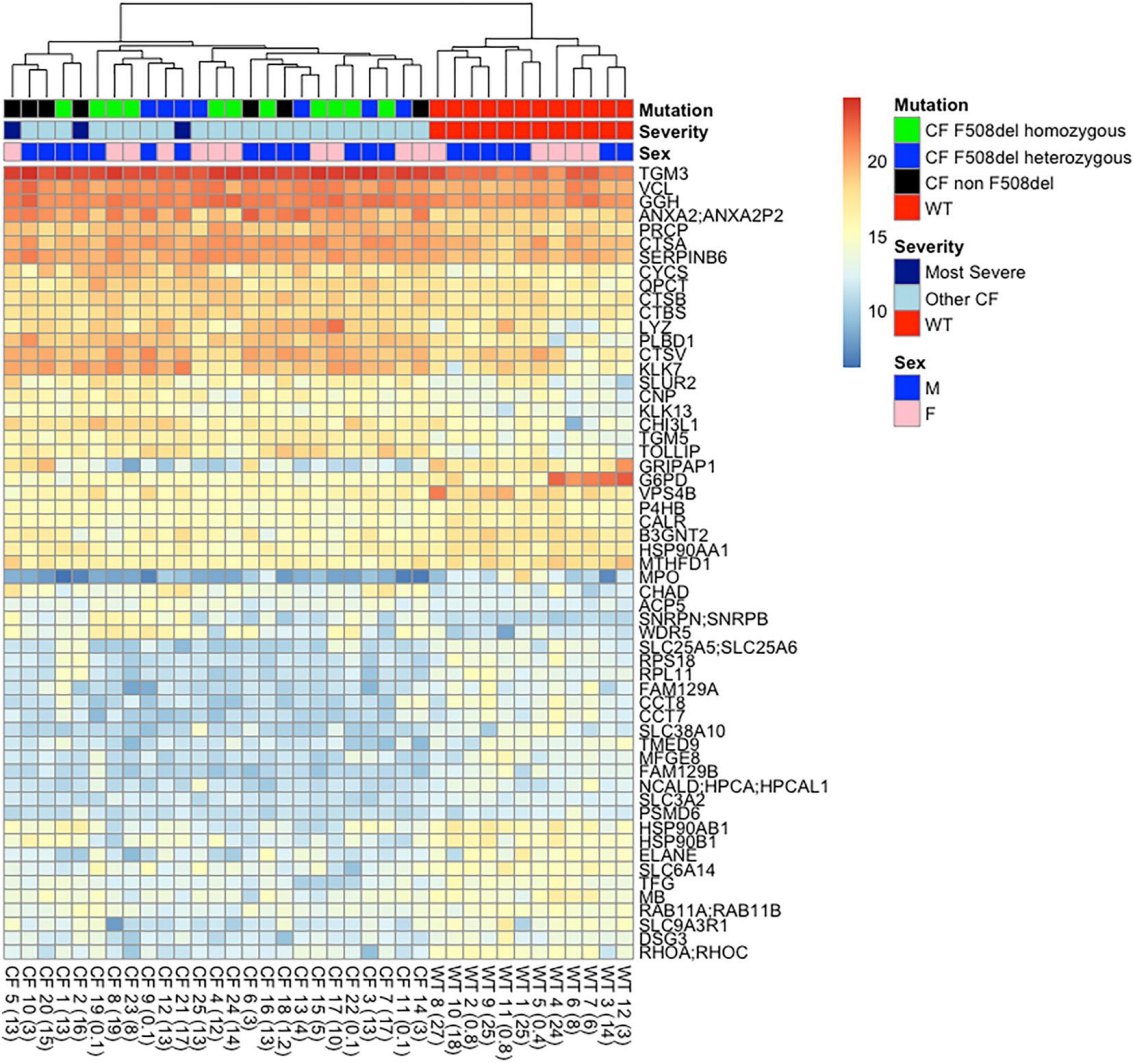
Heat map of differentially expressed proteins between patients with cystic fibrosis (CF) (patients with CF (pwCF)) and non‐CF individuals (WT). Dendrogram is shown according to genetic status (mutation), severity (most severe: ppFEV1 < 50%, chronic sputum colonization with P.aeruginosa or Alcaligenes spp), and sex. Patients' age (in years) is presented after their assigned study number

Fifty‐seven proteins were found differentially abundant between pwCF and WT subjects: 25 were upregulated in CF sweat, and 32 were downregulated (Figure 1, Table 2). Hierarchical analysis confirmed that these proteins clearly separated the pwCF from the WT as shown in Figure 2.

**TABLE 2:** List of differentially expressed proteins in cystic fibrosis (CF) and WT sweat

Protein	P	Q (Benjamini‐Hochberg)	Log fold change (CF ‐ WT)	Expression in CF sweat
TGM3	1.58170503179399e‐09	1.56746968650784e‐06	1.48299833333333	Overexpressed
PLBD1	2.19449916269758e‐08	1.08737433511665e‐05	3.19911266666667	Overexpressed
TGM5	6.43668943899884e‐08	1.96835309247718e‐05	2.09593033333334	Overexpressed
TOLLIP	1.71984520823241e‐06	0.000284061100226386	2.593015	Overexpressed
KLK7	1.99882588608743e‐05	0.00132055763540843	2.80988066666667	Overexpressed
KLK13	1.97919945229845e‐05	0.00132055763540843	1.48745466666667	Overexpressed
CHI3L1	3.01262586508373e‐05	0.00157132222752525	2.6340845	Overexpressed
ANXA2; ANXA2P2	3.88823861049168e‐05	0.00183487831571298	1.92317566666667	Overexpressed
CTSB	4.28375399575546e‐05	0.00192963645899712	1.05712433333333	Overexpressed
CYCS	5.0216366740295e‐05	0.00207351747665135	2.224024	Overexpressed
CNP	6.03328049753096e‐05	0.00239159238922127	1.86662933333333	Overexpressed
VCL	9.66636492911453e‐05	0.00309011859508145	1.04174433333333	Overexpressed
CTBS	0.000100169383365191	0.00310212059109076	0.788042000000001	Overexpressed
CTSV	0.000169602967955179	0.00400182241056147	1.95409066666667	Overexpressed
GGH	0.00020565187231108	0.00463184103318819	0.690882000000002	Overexpressed
SERPINB6	0.000218078527698848	0.00480257379887907	0.856213666666665	Overexpressed
WDR5	0.000259717676076137	0.00547617482960537	2.56654883333333	Overexpressed
CHAD	0.000265784565267186	0.00548734383707879	2.12672166666667	Overexpressed
SNRPN; SNRPB	0.000275203341900658	0.0055658471800725	2.27634633333333	Overexpressed
CTSA	0.000357792614031708	0.0069524015785377	0.989661000000002	Overexpressed
LYZ	0.000355199902106944	0.0069524015785377	2.700541	Overexpressed
SLUR2	0.000419516866382318	0.00784417386009203	2.11938466666667	Overexpressed
PRCP	0.000415002068705056	0.00784417386009203	0.955500666666666	Overexpressed
QPCT	0.000493990855610998	0.00906564699834257	1.365012	Overexpressed
ACP5	0.000527198325269628	0.00932952750611073	1.02944833333333	Overexpressed
FAM129B	7.94491661948408e‐08	1.96835309247718e‐05	−2.0791504	Underexpressed
RHOA; RHOC	2.04192488811702e‐07	4.04709512824794e‐05	−2.41087553333333	Underexpressed
VPS4B	9.60588941329161e‐06	0.00119159328624749	−2.144275	Underexpressed
MPO	1.08217351929641e‐05	0.00119159328624749	−3.42752986666667	Underexpressed
TFG	9.78268215062519e‐06	0.00119159328624749	−1.835101	Underexpressed
ELANE	1.51021002126614e‐05	0.00127553183077237	−2.48330053333333	Underexpressed
GRIPAP1	1.47731114847263e‐05	0.00127553183077237	−4.4502426	Underexpressed
CCT7	1.54453904836211e‐05	0.00127553183077237	−2.04755293333333	Underexpressed
SLC6A14	1.70723579325206e‐05	0.00130143897777907	−2.0631152	Underexpressed
SLC3A2	2.25999565002928e‐05	0.00139978480573689	−0.881463	Underexpressed
RPL11	2.66865246950978e‐05	0.00155566741016717	−1.91789953333333	Underexpressed
P4HB	2.84976534262211e‐05	0.00156895414141028	−0.849515333333335	Underexpressed
G6PD	3.29256052077666e‐05	0.00163146373804484	−3.14162133333333	Underexpressed
TMED9	4.5015504560557e‐05	0.00193958108780487	−1.8657086	Underexpressed
HSP90AB1	6.36883655989668e‐05	0.00242750655032985	−2.41141233333333	Underexpressed
MTHFD1	7.2379130902605e‐05	0.0026565821749808	−1.390594	Underexpressed
CALR	8.12542575545831e‐05	0.00277665411160662	−0.987771333333333	Underexpressed
CCT8	7.98292581291651e‐05	0.00277665411160662	−2.11998133333333	Underexpressed
B3GNT2	8.49078785657414e‐05	0.00280479025528832	−1.879466	Underexpressed
HSP90B1	0.000104409061495325	0.00313543575581414	−2.160834	Underexpressed
HSP90AA1	0.00011554029444987	0.00327144090856632	−1.23706566666667	Underexpressed
SLC38A10	0.000113663277186564	0.00327144090856632	−1.84155993333333	Underexpressed
RPS18	0.000120845307526837	0.00332660277108598	−1.67556433333333	Underexpressed
SLC25A5; SLC25A6	0.000126964753433738	0.00340059650413066	−1.8838592	Underexpressed
MFGE8	0.000134504290978555	0.00350773032525652	−1.57676266666667	Underexpressed
RAB11A; RAB11B	0.000149194674859679	0.00379107494322929	−1.07444733333333	Underexpressed
PSMD6	0.000154226015316499	0.00382094952946626	−0.887891	Underexpressed
DSG3	0.000169543652295849	0.00400182241056147	−1.4409494	Underexpressed
SLC9A3R1	0.000191808840825127	0.00442052468041165	−2.15198906666667	Underexpressed
MB	0.000240247633526712	0.00517576967010808	−1.53099033333333	Underexpressed
FAM129 A	0.000516733099906237	0.00931059094558329	−1.82837733333333	Underexpressed
NCALD; HPCA; HPCAL1	0.000554437456083902	0.0096394301575289	−1.301822	Underexpressed

Abbreviations: CALR, Calreticulin; CHAD, Chondroadherin; CNP, 2',3'‐cyclic nucleotide 3' phosphodiesterase; CTBS, Chitobiase; CTSA, Cathepsin A; CTSB, Cathepsine B; CTSV, Cathepsin V; CYCS, Cytochrome C; ELANE, Elastase, Neutrophil Expressed; GGH, Gamma‐Glutamyl Hydrolase; HPCA, Hippocalcin; LYZ, Lysozyme C; MB, Myoglobin; MPO, Myeloperoxidase; NCALD, Neurocalcin Delta; PRCP, Prolylcarboxypeptidase; QPCT, Glutaminyl‐Peptide Cyclotransferase; RHOA, Ras Homolog Family Member A; RHOC, Ras Homolog Family Member C; SNRPN, small nuclear ribonucleoprotein polypeptide; TFG, Trafficking From ER To Golgi Regulator; VCL, Vinculin.

Sweat proteome profile of F508del homozygous patients was not significantly different from that of compound F508del heterozygotes or patients with other genotypes, as shown in the dendrogram (Figure 2). Furthermore, patients carrying 2 class 1 variants had a similar profile to those carrying at least one class 2 variant. Neither age, nor sex influenced clustering, including in the infants. We thus performed statistical analysis on the overall pwCF cohort to increase the significance of our results.

Cystic Fibrosis sweat proteome was characterized by an increased level in (i) proteases (Kallikrein 7 and 13, Phospholipase B domain containing 1, Cathepsin A L2 and B, Lysosomal Pro‐X carboxypeptidase, neuraminidase); (ii) proinflammatory proteins (Annexin A2, Chitinase‐3‐like protein 1); (iii) cytochrome c; (iv) Transglutaminases 3 and 5; and (v) Toll‐interacting protein (TOLLIP). This contrasted with decrease of Elastase 2, an epidermal protease, FAM129 B, an antioxidative protein and Desmoglein‐3. Finally, 2 proteins known to interfere with CFTR biology were underexpressed (Sodium‐hydrogen antiporter 3 regulator 1 [NHERF1] and Solute Carrier Family 6 Member 14, SLC6A14).

The severity of the disease, based on a ppFEV1 below 50%, and/or chronic colonization with *Pseudomonas*.*aeruginosa* or *Alcaligenes spp* did not modify the proteome profile.

## 5 DISCUSSION

This study is to our knowledge the first report of CF sweat proteome. We were able to yield nearly 1000 proteins, in as low as 20 μL of sweat thanks to an optimized protocol with high‐resolution LC–MS/MS acquisition. To our knowledge, a similar depth of proteome coverage was only reported in 2 previous studies in healthy adults and using larger volumes of sweat. Yu et al performed the analysis on 10 ml pooled samples collected from absorbing tissue pads at different body sites after exercise while Burat et al used a mean volume of 70 μL of sweat.^10^, ^11^ We used 20 μL of leftover sweat sample collected for the sweat test. Usually at least 50 μL of sweat are collected for sweat test and around 20 μL are used for sweat Cl^−^ concentration evaluation. Our results demonstrate that the remainder aliquot of a diagnostic sweat test can be used reliably for sweat proteomics, including in infants as young as 1 month of age.

A large range of proteases and their respective inhibitors, antimicrobial peptides and effectors of skin innate immunity have already been reported in sweat of healthy subjects^10^, ^11^, ^12^, ^13^, ^14^ or in specific diseases such as schizophrenia or tuberculosis.^15^, ^16^ Detection of other proteins such as cytoskeletal proteins or proteins involved in oxidative stress, unfolded protein response, Endoplasmic Reticulum stress, proteasome and cancellous metabolism pathways points to the diversity of sweat biology.^11^, ^17^, ^18^, ^19^, ^20^ Although some studies report a correlation between blood versus sweat concentration for specific biomarkers such as glucose,^16^, ^21^ the literature shows mixed results for others^22^ and relatively few proteins have yet been fully assessed for blood versus sweat concentration correlation.

As in previous studies, we did not report any strong influence of sex or age, even in 1 month old babies. Nor did we observe any influence of disease severity based on genetics or lung disease pattern, although the very limited number of patients with severe disease precludes a definitive conclusion.

Our results add additional insights in sweat physiology by showing an unbalanced proteolytic and proinflammatory activity of this biofluid, in pwCF. A number of proteases were upregulated, including Kallikreins 7 and 13. These epidermal‐specific serine proteases activate a proteolytic cascade, generating skin inflammation and ultimately skin physical barrier impairment, as already reported in the Netherton Syndrome or Atopic Dermatitis.^23^ Annexin A2 forms a complex with S100A10 involved in a large variety of pathophysiological processes, including activation of metalloprotease induced inflammation.^24^ Other regulators of innate immunity were also significantly upregulated, such as Chitinase‐3‐like protein (CHI3L1) and TOLLIP. CHI3L1 reflects induction of T‐helper cell type 2, IL‐13‐induced inflammation, and macrophage activation.^25^ Interestingly, this protein is also elevated in sputum and serum of CF patients with lung disease.^26^

Toll‐interacting protein is an intermediate in interleukin (IL)‐1 signalling involved in the Toll‐like receptor mediated inflammation and triggers the nuclear factor (NF)‐κB and mitogen‐activated protein kinase signalling.^27^ All these proinflammatory signalling cascades may be associated with an increased oxidative status, as assessed by the increase in Cytochrome C, a mitochondrial protein involved in the electron transport system and oxidative phosphorylation. This may be potentiated by the decrease of the antioxidative protein FAM129B6.^28^

Our results also suggest a defect in the integrity of the skin barrier in pwCF. Indeed, downregulation of elastase 2 is anticipated to decrease the cleavage of filaggrin, a protein which plays a key role in the integrity of the epidermal skin barrier through its end products.^29^, ^30^ Absence of filaggrin in null carriers predisposes to ichthyosis vulgaris, and/or atopic dermatitis.^31^, ^32^ Downregulation of Desmoglein 3, a component of desmosomes, should reinforce the skin barrier defect by decreasing cell to cell junction.^33^ Finally, transglutaminases overexpression may perturb the formation of the outer layer of the epidermis by altering cross‐linking of keratins and structural proteins.^34^

All these defects should result in altered skin barrier function and favour increased water content when the skin is immerged in water, one of the main features observed in AKP.^35^ Indeed, although the pathogenesis of AKP is not fully understood, it is assumed to be related to hydropic changes of the horny layer and the excessive electrolyte content of CF sweat which moistens the epidermis obviously plays an additional role.^34^ The observation that CFTR function restoration by ivacaftor, a highly efficient CFTR modulator correcting Cl^−^ channel activity, improves AKP symptoms provides clinical evidence that this condition is related to CFTR dysfunction.^5^ Interestingly, in our data set, we also show a defective expression of 2 proteins known to modulate the expression and function of CFTR at the apical membrane: NHERF, a protein known to anchor CFTR at the membrane and SLC26A14, an amino‐acid transporter involved in cGMP‐mediated F508del‐CFTR channel activity.^35^, ^36^

Our study is the first report of human sweat proteome in pwCF.

Altogether, our results, based on a deep proteomic evaluation, provide evidence for an unbalanced proteolytic and proinflammatory activity of CF sweat, and alteration of signalling pathways involved in the integrity of the epidermal skin barrier. This CF‐specific profile provides novel insight for AKP physiopathology and CFTR biology and may be used in the context of personalized medicine in CF.

## AUTHOR CONTRIBUTIONS

**Matthieu Cornet**: Formal analysis (equal); Validation (equal); Visualization (equal); Writing – original draft (equal). **Thao Nguyen‐Khoa**: Conceptualization (equal); Data curation (equal); Investigation (equal). **Mairead Kelly‐Aubert**: Investigation (equal); Writing – review & editing (equal). **Vincent Jung**: Data curation (equal); Formal analysis (equal); Investigation (equal). **Frederique Chedevergne**: Data curation (equal); Investigation (equal). **Muriel Le Bourgeois**: Data curation (equal); Investigation (equal). **Laura Aoust**: Data curation; Investigation (equal). **Kevin Roger**: Formal analysis; Visualization; Investigation (equal). **Chiara Guerrera**: Conceptualization (equal); Data curation; Investigation; Methodology; Project administration (equal); Resources (equal); Supervision (equal); Writing – original draft; Writing – review & editing (equal). **Isabelle Sermet‐Gaudelus**: Conceptualization (equal); Funding acquisition (equal); Investigation; Methodology; Project administration (equal); Resources (equal); Supervision (equal); Writing – original draft; Writing – review & editing (equal).

## CONFLICT OF INTEREST

Isabelle Sermet‐Gaudelus has been awarded a grant from Vertex Therapeutics (Vertex Innovation Award). She participates in Vertex Therapeutics, Eloxx Therapeutics and Proteostasis Therapeutics scientific boards.

## ETHICS STATEMENT

All participants signed an informed consent form for sweat collection and analysis (no. Eudract 2016‐A00309‐42; Comité de Protection des Personnes IDF2).

## Data Availability

Data available on request from the authors.

## References

[1] Elborn JS . Cystic fibrosis. Lancet Lond Engl. 2016;388(10059):2519–31. 10.1016/s0140-6736(16)00576-6 27140670

[2] Alexopoulos A , Chouliaras G , Kakourou T , Dakoutrou M , Nasi L , Petrocheilou A , et al. Aquagenic wrinkling of the palms after brief immersion to water test as a screening tool for cystic fibrosis diagnosis. J Eur Acad Dermatol Venereol JEADV. 2021;35(8):1717–24. 10.1111/jdv.17312 33914973

[3] Arkin LM , Flory JH , Shin DB , Gelfand JM , Treat JR , Allen J , et al. High prevalence of aquagenic wrinkling of the palms in patients with cystic fibrosis and association with measurable increases in transepidermal water loss. Pediatr Dermatol. 2012;29(5):560–6. 10.1111/j.1525-1470.2011.01708.x 22471628

[4] Berk DR , Ciliberto HM , Sweet SC , Ferkol TW , Bayliss SJ . Aquagenic wrinkling of the palms in cystic fibrosis: comparison with controls and genotype‐phenotype correlations. Arch Dermatol. 2009;145(11):1296–9. 10.1001/archdermatol.2009.260 19917960

[5] Jacobi E , Solomon M , Avolio J , Shaw M , Gonska T , Ratjen F , et al. Aquagenic wrinkling of the palms in cystic fibrosis patients treated with ivacaftor. J Cyst Fibros Off J Eur Cyst Fibros Soc. 2022;21(2):e102–5. 10.1016/j.jcf.2022.01.005 35063397

[6] Sermet‐Gaudelus I , Nguyen‐Khoa T , Hatton A , Hayes K , Pranke I . Sweat chloride testing and nasal potential difference (NPD) are primary outcome parameters in treatment with cystic fibrosis Transmembrane conductance regulator (CFTR) modulators. J Pers Med. 2021;11(8):729. 10.3390/jpm11080729 34442373 PMC8398324

[7] Cox J , Mann M . MaxQuant enables high peptide identification rates, individualized p.p.b.‐range mass accuracies and proteome‐wide protein quantification. Nat Biotechnol. 2008;26(12):1367–72. 10.1038/nbt.1511 19029910

[8] Nguyen‐Khoa T , Hatton A , Drummond D , Aoust L , Schlatter J , Martin C , et al. Reclassifying inconclusive diagnosis for Cystic Fibrosis with new generation sweat test. Eur Respir J. 2022;60(2):2200209. 10.1183/13993003.00209-2022 35777769

[9] Duguépéroux I , De Braekeleer M . The CFTR 3849+10kbC‐>T and 2789+5G‐>A alleles are associated with a mild CF phenotype. Eur Respir J. 2005;25(3):468–73. 10.1183/09031936.05.10100004 15738290

[10] Yu Y , Prassas I , Muytjens CMJ , Diamandis EP . Proteomic and peptidomic analysis of human sweat with emphasis on proteolysis. J Proteomics. 2017;155:40–8. 10.1016/j.jprot.2017.01.005 28095327

[11] Burat B , Reynaerts A , Baiwir D , Fleron M , Eppe G , Leal T , et al. Characterization of the human eccrine sweat proteome‐A focus on the biological variability of individual sweat protein profiles. Int J Mol Sci. 2021;22(19):10871. 10.3390/ijms221910871 34639210 PMC8509809

[12] Csősz É , Emri G , Kalló G , Tsaprailis G , Tozser J . Highly abundant defense proteins in human sweat as revealed by targeted proteomics and label‐free quantification mass spectrometry. J Eur Acad Dermatol Venereol JEADV. 2015;29(10):2024–31. 10.1111/jdv.13221 26307449 PMC4583350

[13] Harshman SW , Pitsch RL , Smith ZK , O’Connor ML , Geier BA , Qualley AV , et al. The proteomic and metabolomic characterization of exercise‐induced sweat for human performance monitoring: a pilot investigation. PLoS One. 2018;13(11):e0203133. 10.1371/journal.pone.0203133 30383773 PMC6211630

[14] Katchman BA , Zhu M , Blain Christen J , Anderson KS . Eccrine sweat as a biofluid for profiling immune biomarkers. Proteomics Clin Appl. 2018;12(6):e1800010. 10.1002/prca.201800010 29882373 PMC6282813

[15] Raiszadeh MM , Ross MM , Russo PS , Schaepper MA , Zhou W , Deng J , et al. Proteomic analysis of eccrine sweat: implications for the discovery of schizophrenia biomarker proteins. J Proteome Res. 2012;11(4):2127–39. 10.1021/pr2007957 22256890 PMC3703649

[16] Adewole OO , Erhabor GE , Adewole TO , Ojo AO , Oshokoya H , Wolfe LM , et al. Proteomic profiling of eccrine sweat reveals its potential as a diagnostic biofluid for active tuberculosis. Proteomics Clin Appl. 2016;10(5):547–53. 10.1002/prca.201500071 26948146

[17] Baechle D , Flad T , Cansier A , Steffen H , Schittek B , Tolson J , et al. Cathepsin D is present in human eccrine sweat and involved in the postsecretory processing of the antimicrobial peptide DCD‐1L. J Biol Chem. 2006;281(9):5406–15. 10.1074/jbc.m504670200 16354654

[18] Murakami M , Ohtake T , Dorschner RA , Gallo RL , Schittek B , Garbe C . Cathelicidin anti‐microbial peptide expression in sweat, an innate defense system for the skin. J Invest Dermatol. 2002;119(5):1090–5. 10.1046/j.1523-1747.2002.19507.x 12445197

[19] Park JH , Park GT , Cho IH , Sim SM , Yang JM , Lee DY . An antimicrobial protein, lactoferrin exists in the sweat: proteomic analysis of sweat. Exp Dermatol. 2011;20(4):369–71. 10.1111/j.1600-0625.2010.01218.x 21366701

[20] Schittek B , Hipfel R , Sauer B , Bauer J , Kalbacher H , Stevanovic S , et al. Dermcidin: a novel human antibiotic peptide secreted by sweat glands. Nat Immunol. 2001;2(12):1133–7. 10.1038/ni732 11694882

[21] Moyer J , Wilson D , Finkelshtein I , Wong B , Potts R . Correlation between sweat glucose and blood glucose in subjects with diabetes. Diabetes Technol Ther. 2012;14(5):398–402. 10.1089/dia.2011.0262 22376082

[22] Baker LB . Physiology of sweat gland function: the roles of sweating and sweat composition in human health. Temp Austin Tex. 2019;6(3):211–59. 10.1080/23328940.2019.1632145 PMC6773238 31608304

[23] Kasparek P , Ileninova Z , Zbodakova O , Kanchev I , Benada O , Chalupsky K , et al. KLK5 and KLK7 ablation fully rescues lethality of Netherton syndrome‐like phenotype. PLoS Genet. 2017;13(1):e1006566. 10.1371/journal.pgen.1006566 28095415 PMC5283769

[24] Dallacasagrande V , Hajjar KA . Annexin A2 in inflammation and host defense. Cells. 2020;9(6):E1499. 10.3390/cells9061499 PMC7348701 32575495

[25] Kwak EJ , Hong JY , Kim MN , Kim SY , Kim SH , Park CO , et al. Chitinase 3‐like 1 drives allergic skin inflammation via Th2 immunity and M2 macrophage activation. Clin Exp Allergy J Br Soc Allergy Clin Immunol. 2019;49(11):1464–74. 10.1111/cea.13478 31397016

[26] Leonardi S , Parisi GF , Capizzi A , Manti S , Cuppari C , Scuderi MG , et al. YKL‐40 as marker of severe lung disease in cystic fibrosis patients. J Cyst Fibros Off J Eur Cyst Fibros Soc. 2016;15(5):583–6. 10.1016/j.jcf.2015.12.020 26778616

[27] Burns K , Clatworthy J , Martin L , Martinon F , Plumpton C , Maschera B , et al. Tollip, a new component of the IL‐1RI pathway, links IRAK to the IL‐1 receptor. Nat Cell Biol. 2000;2(6):346–51. 10.1038/35014038 10854325

[28] Cheng KC , Lin RJ , Cheng JY , Wang SH , Yu JC , Wu JC , et al. FAM129B, an antioxidative protein, reduces chemosensitivity by competing with Nrf2 for Keap1 binding. EBioMedicine. 2019;45:25–38. 10.1016/j.ebiom.2019.06.022 31262713 PMC6642435

[29] Bonnart C , Deraison C , Lacroix M , Uchida Y , Besson C , Robin A , et al. Elastase 2 is expressed in human and mouse epidermis and impairs skin barrier function in Netherton syndrome through filaggrin and lipid misprocessing. J Clin Invest. 2010;120(3):871–82. 10.1172/jci41440 20179351 PMC2827963

[30] Sandilands A , Sutherland C , Irvine AD , McLean WHI . Filaggrin in the frontline: role in skin barrier function and disease. J Cell Sci. 2009;122(Pt 9):1285–94. 10.1242/jcs.033969 19386895 PMC2721001

[31] Palmer CNA , Irvine AD , Terron‐Kwiatkowski A , Zhao Y , Liao H , Lee SP , et al. Common loss‐of‐function variants of the epidermal barrier protein filaggrin are a major predisposing factor for atopic dermatitis. Nat Genet. 2006;38(4):441–6. 16550169 10.1038/ng1767

[32] Weidinger S , Illig T , Baurecht H , Irvine AD , Rodriguez E , Diaz‐Lacava A , et al. Loss‐of‐function variations within the filaggrin gene predispose for atopic dermatitis with allergic sensitizations. J Allergy Clin Immunol. 2006;118(1):214–9. 10.1016/j.jaci.2006.05.004 16815158

[33] Bonné S , Gilbert B , Hatzfeld M , Chen X , Green KJ , van Roy F . Defining desmosomal plakophilin‐3 interactions. J Cell Biol. 2003;161(2):403–16. 10.1083/jcb.200303036 12707304 PMC2172904

[34] Rongioletti F , Tomasini C , Crovato F , Marchesi L . Aquagenic (pseudo) keratoderma: a clinical series with new pathological insights. Br J Dermatol. 2012;167(3):575–82. 10.1111/j.1365-2133.2012.11003.x 22512866

[35] Seidler U , Singh AK , Cinar A , Chen M , Hillesheim J , Hogema B , et al. The role of the NHERF family of PDZ scaffolding proteins in the regulation of salt and water transport. Ann N Y Acad Sci. 2009;1165(1):249–60. 10.1111/j.1749-6632.2009.04046.x 19538313

[36] Ruffin M , Mercier J , Calmel C , Mesinele J , Bigot J , Sutanto EN , et al. Update on SLC6A14 in lung and gastrointestinal physiology and physiopathology: focus on cystic fibrosis. Cell Mol Life Sci CMLS. 2020;77(17):3311–23. 10.1007/s00018-020-03487-x 32166393 PMC7426304

